# Supplementary material for: Characterization of Diarreaghenic Escherichia coli Strains Isolated from Healthy Donors, including a Triple Hybrid Strain
Source: Antibiotics (Basel). 2022 Jun 21;11(7):833. doi: 10.3390/antibiotics11070833 (PMC9312309; doi:10.3390/antibiotics11070833)
Supplement: Supplementary file 1 [file antibiotics-11-00833-s001.zip › antibiotics-1778457-supplementary.pdf]

Supplementary Table S1. Specific oligonucleotides used in this study

Gene	Sequence (5´-3´)	Amplicon size	Reference
chuA	F: atggtaccggacgaaccaac<br>R: tgccgccagtaccaaagaca	288bp	
yjaA	F: caaacgtgaagtgtcaggag<br>R: aatgcgttcctcaacctgtg	211bp	
TspE4. C2	F: cactattcgtaaggtcatcc<br>R: agtttatcgctgcgggtcgc	152bp	
arpA	F: aacgctattcgccagcttgc<br>R: tctccccataccgtacgcta	400bp	[37]
arpA	F: gattccatcttgtcaaaatatgcc<br>R: gaaaagaaaaagaattcccaagag	301bp	
trpA	F: gaaaagaaaaagaattcccaagag<br>R: agttttatgcccagtgcgag	219bp	
trpA	F: cggcgataaagacatcttcac<br>R: gcaacgcggcctggcggaag	489bp	
ybbW	F: gtgattggcaaaatctggccg<br>R: catactggcaatcagtacgcc	667bp	[38,39]
bfpA	F: aatggtgcttgcgcttgctgc<br>R: gccgctttatccaacctggta	326bp	[40]
eaeA	F: caggtcgtcgtgtctgctaaa<br>R: tcagcgtggttggatcaacct	570bp	[41]
pEAF	F: cagggtaaaagaaagatgata<br>R: tatggggaccatgtattatca	399bp	[42]
stx1	F: tttacgatagacttctcgac<br>R: cacatataaattatttcgctc	227bp	[43]
stx2	F: cccagtcacgacgttgta<br>R: tatactatcgtgcctttcca	460bp	[44]
elt	F: ggcgacagattataccgtgc<br>R: cggtctctatattccctgtt	450bp	[45]
estI	F: ttaatagcacccggtacaagcagg<br>R: cttgactcttcaaaagagaaaattac	93bp	[46]
staII	F: ttgtctttttcacctttccc<br>R: acaagcaggattacaacaca	93bp	[44]
daaE	F: tgactgtgaccgaagagtgc<br>R: ttagttcgtccagtaaccccc	380bp	[47]
pCVD432	F: ctggcgaaagactgtatcat<br>R: caatgtatagaaatcgctgtt	630bp	[44]
iaI	F: ctggatggtatggtgagg<br>R: ggaggccaacaattatttcc	320bp	[44]
CTX-M1&8	F: tgtgcagyaccagtaargykatg	583bp	[50]

	R:tarrtsaccagaayvagcggc	
CTX-M2	F:cgagtggcagtaccagtaagg<br>R:cgatatcgttggtggtgc	540bp
CTX-M9	F:atggtgacaaagagagtgcaa<br>R:aatatcattggtggtgccgtag	747bp
CTX-M151	F:gcggccatgataggtacg<br>R:aaagtaagtcacaataaccagcg	786bp
TEM	F:caacattttcgtgtcgccc<br>R:gcttaatcagtgaggcacc	844bp
SHV	F:tattatctccctgttagcca<br>R:cgctctgctttgttattc	783bp
